# Characterization of the Temporal Trends in the Rate of Cattle Carcass Condemnations in the US and Dynamic Modeling of the Condemnation Reasons in California With a Seasonal Component

**DOI:** 10.3389/fvets.2018.00087

**Published:** 2018-06-19

**Authors:** Sara Amirpour Haredasht, Gema Vidal, Anita Edmondson, Dale Moore, Noelia Silva-del-Río, Beatriz Martínez-López

**Affiliations:** ^1^Center for Animal Disease Modeling and Surveillance (CADMS), Department of Medicine & Epidemiology, School of Veterinary Medicine, University of California, Davis, Davis, CA, United States; ^2^Animal Health Branch, California Department of Food and Agriculture (CDFA), Sacramento, CA, United States; ^3^Department of Veterinary Clinical Sciences, College of Veterinary Medicine, Washington State University, Pullman, WA, United States; ^4^Department of Population Health and Reproduction, School of Veterinary Medicine, University of California, Davis, CA, United States

**Keywords:** syndromic surveillance, dynamic harmonic regression, slaughterhouse, early detection, warning system

## Abstract

Based on the 2016 National Cattlemen's Beef Association statistics, the cattle inventory in the US reached 93.5 million head, from which 30.5 million were commercial slaughter in 2016. California ranked fourth among all the US states that raise cattle and calves, with 5.15 million head and approximately 1.18 million slaughtered animals per year. Approximately 0.5% of cattle carcasses in the US are condemned each year, which has an important economic impact on cattle producers.In this study, we first described and compared the temporal trends of cattle carcass condemnations in all the US states from Jan-2005 to Dec-2014. Then, we focused on the condemnation reasons with a seasonal component in California and used dynamic harmonic regression (DHR) models both to model (from Jan-2005 to Dec-2011) and predict (from Jan-2012 to Dec-2014) the carcass condemnations rate in different time horizons (3 to 12 months).Data consisted of daily reports of 35 condemnation reasons per cattle type reported in 684 federally inspected slaughterhouses in the US from Jan-2005 to Dec-2014 and the monthly slaughtered animals per cattle type per states. Almost 1.5 million carcasses were condemned in the US during the 10 year study period (Jan 2005-Dec 2014), and around 40% were associated with three condemnation reasons: malignant lymphoma, septicemia and pneumonia. In California, emaciation, eosinophilic myositis and malignant lymphoma were the only condemnation reasons presenting seasonality and, therefore, the only ones selected to be modeled using DHRs. The DHR models for Jan-2005 to Dec-2011 were able to correctly model the dynamics of the emaciation, malignant lymphoma and eosinophilic myositis condemnation rates with coefficient of determination ($R_{t}^{2}$) of 0.98, 0.87 and 0.78, respectively. The DHR models for Jan-2012 to Dec-2014 were able to predict the rate of condemned carcasses 3 month ahead of time with mean relative prediction error of 33, 11, and 38%, respectively. The systematic analysis of carcass condemnations and slaughter data in a more real-time fashion could be used to identify changes in carcass condemnation trends and more timely support the implementation of prevention and mitigation strategies that reduce the number of carcass condemnations in the US.

## Introduction

The meat industry is the largest segment of US agriculture based on economic land use and environmental impact. The United States Department of Agriculture (USDA) reported that in 2015, more than 28,296,403 cattle were commercially slaughtered with a total carcass weight of 3,276,367,000 pounds (1). From those, 141,450 carcasses were condemned, representing approximately 0.5% of the total cattle carcasses produced in the US. Similar results were provided from 2003 through 2007 (i.e., 5 years), where more than 163 million cattle (excluding bob veal, veal, and heavy calves) arrived at USDA-inspected slaughter facilities and 769,339 (0.47%) were condemned at either antemortem or postmortem inspection (2).

Slaughter data are valuable sources of information because they include both demographic (age, type of cattle) and health related (reason for condemnation) factors. The systematic analysis of slaughter data can help identify the most important condemnation reasons, characterize seasonality and emerging spatio-temporal trends, and provide the foundations of syndromic surveillance systems and early-detection of outbreaks. Two European countries and Canada are currently using (e.g., Switzerland; 3) or plan to use [e.g., France, (4) and Canada (5)] data collected during meat inspection for syndromic surveillance of animal health. For example, the French Ministry of Agriculture started the Nergal-Abattoir project to collect data in real-time during the slaughtering process for timely surveillance and risk factor analysis (4). However, there are very few published studies using slaughterhouse data for outbreak investigation or syndromic surveillance in the US. The study by White and Moore (2) highlighted the need to understand condemnation reasons for producer education and early intervention by veterinarians. Studies by Kaneene et al. (6) and Humphrey et al. (7) used US slaughterhouse data to trace cattle to the herd of origin after detection and confirmation of bovine tuberculosis. Their main concern was the failure to trace back some bovine tuberculosis-positive animals. Other studies have shown the value of using slaughter data for example to early detect erysipelas outbreaks in the US swine industry (8) or predict increases in transport losses of swine in-transit and just prior to slaughter (9).

Real-time access to slaughter data and a better knowledge of temporal trends of the number of reported condemned carcasses in slaughterhouses in the US could be used to identify times, areas and cattle types with higher than expected numbers of carcass condemnations that may be associated with specific management practices, adverse climatic events, or emerging syndromes and new diseases [e.g., (10)]. This can help to target the allocation of risk based strategies or the implementation of education and preventive management practices in those high risk areas that are particularly affected by specific condemnation reasons.

However, before the interpretation of results suggesting an increase or decrease in the number of carcass condemnation cases it is important to understand and identify seasonal trends or components, which are considered to be normal variations of the time series, as without correcting for those trends the results may be biased (5). A dynamic harmonic regression (DHR) model can be regarded as a time-series component model, where the phases and amplitude of seasonal and cyclic components are represented by “dynamic” or “time-varying” parameters (TVP), reflecting relevant changes in the evolution of the patterns of different carcass condemnation reasons. This can be contrasted with conventional seasonal auto regression, where it is assumed that the amplitude and phase of the periodic components are time invariant (11). The DHR model structures can be defined by autoregressive spectrum analysis thus avoiding the subjective choices of frequencies in the seasonal and cyclic components (12).

In this study, our first objective was to describe and compare the temporal trends of reported condemnation rates in slaughterhouses in California with those in the US from Jan-2005 to Dec-2011. The second objective was to identify those condemnation reasons with a seasonal component using autoregressive spectrum analysis. A third objective was to evaluate if DHR models could predict the rate of carcass condemnations that showed seasonality in future time periods (i.e., from Jan-2012 to Dec-2014) using the seasonal component of the time series. All this information will be useful to increase awareness of producers about the main reasons of carcass condemnations in their specific state and can help them to prioritize the management practices and risk mitigation strategies that could be implemented to minimize future carcass condemnations.

## Materials and Methods

### Data

**Figure 1:**
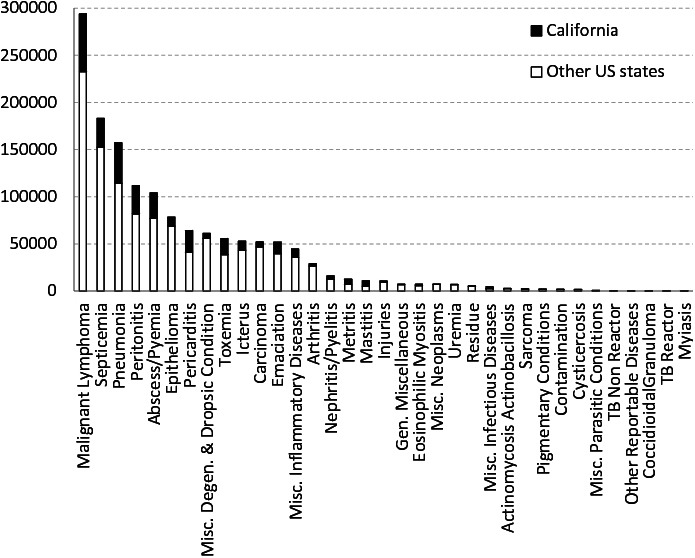
Total number of carcass condemnations per reason in slaughterhouses in California (black) and in other US states (white) from Jan-2005 to Dec-2014.

Data on cattle condemnation reasons were obtained with the Freedom of Information Act from the Food Safety and Inspection Service (FSIS) of the United States Department of Agriculture (USDA), the agency responsible for slaughter conditions and meat inspection. The FSIS inspectors across the US followed similar practices/protocols and had similar training (13). Data consisted of daily reports of condemned carcasses per cattle type and condemnation reasons reported in 684 federally inspected slaughterhouses in US from Jan-2005 to Dec-2014, including 29 slaughterhouses located in California. For each animal, the database contained: condemned reason (*n* = 35; Figure 1), cattle ID number, slaughter date, slaughterhouse’s name (*n* = 684) and state (*n* = 49). Slaughtered cattle types were beef cow, bob veal, bull/stag, dairy cow, formula-fed veal, heavy calf, heifer, non-formula-fed veal and steer (14). The monthly number of cattle sent to slaughterhouse per state and per cattle type from Jan-2005 to Dec-2014 was also provided by FSIS.

### Dynamic Harmonic Regression Model

The DHR model was conducted using the monthly rate of reported carcass condemnations for each condemnation reason (i.e., the number of the carcass condemned for a particular reason divided by the number of the slaughtered cattle per month and per type of cattle) in slaughterhouses in California from Jan-2005 to Dec-2014. The number of condemned carcasses in California slaughterhouses were reported daily but aggregated on a monthly basis to compute monthly rates because the number of slaughtered animals were only available per month. In general, the theoretical seasonal periodic components are 12, 6, 4, 3, 2.4 and 2 months per cycle (i.e., the seasonal periodicity is defined as *T*/*j *where *j* = 1, 2,..., 6 and *T* = 12 is the duration of one year with 12 samples as suggested by Taylor et al. (12). However, rather than rely on this theoretical periodicity, we identified the most significant periods in each of the time series of carcass condemnations using an autoregressive spectrum (15). The order of the autoregressive spectrum was selected using the Akaike Information Criteria (16). We considered the rates of carcass condemnation by each specific reason to have a seasonal component if it has a pronounced peak value of equal or close to 12 months per cycle and has at least another dominant peak with the value close to one of the theoretical seasonal periodic component.

For those condemnations reasons that showed evidence of periodicity based on autoregressive spectrum a DHR model was used to quantify their seasonality (15). The DHR approach has been used to quantify periodic components of a time series and understand seasonal fluctuations and temporal trends of time series data in previous studies (e.g., 11, 17, 18). The DHR model is defined as follows (12):

(1) Y(t)=L(t)+Q(t)+e(t)

where Y(t) is the observed time series (i.e., rate of carcass condemnations in California slaughterhouses); L(t) is a trend or low frequency component; e(t) is a “residual” component, normally defined for analytical convenience as a normally distributed Gaussian sequence with zero mean value and variance $\sigma^{2}$ (i.e., discrete-time white noise); and *Q*(*t*) is a cyclical term defined as follows:

(2) $$Q\left(t\right)=\sum_{i=1}^{n=7}\left\{\alpha_{i}\left(t\right)cos\left(f_{i}2\pi t\right)+\beta_{i}\left(t\right)sin\left(f_{i}2\pi t\right)\right\}$$

In this model, $\alpha_{i}\left(t\right)$ and $\beta_{i}\left(t\right)$ are stochastic time variable parameters and, $f_{i}=\frac{1}{T/i}$,* i* = 1,2,..., *n* are the fundamental and harmonic frequencies associated with the periodicity (T/i) in the series (as mentioned before, T is the duration of one year with 12 samples).

The trend components $L\left(t\right)$ in equation 1 and time variable parameters in equation 2 [α(t) and β(t)] were all represented using a general random walk model. The general random walk family of models includes the well-known Random Walk and Integrated Random Walk models (12). We limited analyses to these two types of models.

In particular, each of the time variable parameters in this analysis [$\alpha_{i}\left(t\right)$ and $\beta_{i}\left(t\right),\;i=1,\;2,\;\ldots ,\;n$] were represented as a Random Walk process of the form:

(3) $$\alpha \left(t\right)=\alpha \left(t-1\right)+\eta_{\alpha i}\left(t\right)$$

(4) $$\beta \left(t\right)=\beta \left(t-1\right)+\eta_{\beta i}\left(t\right)\;\;\;$$

where $\eta_{i}(t)$ is an error with zero mean for either αorβ. In this manner, the estimation algorithm is instructed that the parameter in question(αandβ) is a stochastic variable that is likely to change by an unknown but small amount over each sampling interval (here, one month), within the stochastic limits imposed by the variance $\sigma_{\eta_{i}}(t)$ (12).

The trend component $L\left(t\right)$ in equation 1 was modelled as an Integrated Random Walk of the form:

(5) L(t)=L(t−1)+l(t−1)

$$l\left(t\right)=l\left(t-1\right)+\eta_{L}\left(t\right)$$

where l(t) represents the "slope" of the trend, $\eta_{L}\left(t\right)$ is a zero mean, white noise input. Changes in trends [L(t)] of the reported reasons for carcass condemnation in cattle slaughterhouses in California and US were evaluated by calculating the slope of each of the time series. The trend is calculated using an integrated random walk model with noise-variance ratio (NVR) of 0 (equation 5). If the calculated slope is positive, the rate of condemned carcasses is increasing. A negative slope indicates a general decrease in the rate of condemned carcasses. The larger the absolute number of the slope, the steeper the changes in the rate of condemned carcasses.

The variances of the white noise, $\eta_{i}\left(t\right)$, in the Random Walk and Integrated Random Walk models (equation 3, 4 and 5) are critical in estimating the time variable parameters and need to be optimized against the data. The ratio of these variances to the variance of the residual e(t) (equation 1) is called the noise-variance ratio (12).

The DHR algorithm estimates the time variable parameters using the Kalman Filter (19). First, we used DHR model to simulate the dynamics of the rates of carcass condemnations from Jan-2005 to Dec-2011. Then, we used the DHR model to predict the rates of carcass condemnations from Jan-2012 to Dec-2014. The DHR parameters obtained in the simulation (from Jan-2005 to Dec-2011) were used to predict the carcass condemnations for the time period Jan-2012 to Dec-2014. Analyses were conducted using the Captain toolbox^®^ version 7.5 (12) in Matlab^® ^(version 2016a, MathWorks Inc., Natick, MA).

The resulting models were evaluated by the coefficient of determination ($R_{t}^{2}$) (20), which indicates the goodness of fit between the regression line (simulated results) and the observed rate of carcass condemnations.

### Evaluation of the Predicted Capability of the DHR Models

The goodness of fit of the forecasting models were calculated by using the mean relative prediction error (MRPE) defined as (21):

(6) $$MRPE\left(\%\right)=\frac{1}{n}\sum_{t=1}^{n}\sqrt{{\left[\frac{Y\left(t\right)-\hat{Y}\left(t\right)}{Y\left(t\right)}\right]}^{2}}\times 100$$

where Y(t),i=1,2,3...n are the rate of cattle carcass condemnations reported in California slaughterhouses from Jan-2012 to Dec-2014, $\hat{Y}$*(t)* are the last predicted rate of cattle carcass condemnations at the time to which forecasts were made (3 to 12 month) and trepresents discrete-time instants with a measurement interval of 1 month. For each sample of the data set (i.e., every month from Jan-2012 onwards), we estimated the model parameters and generated the 3-month-ahead prediction. To calculate the MRPE for each predictive horizon (3 up to 12 months ahead), we followed the same processes. MRPE values were calculated for all samples, and then averaged for the whole data set. The best model was considered to be the one with the lowest MRPE value. MRPE has been used by several authors and considered a good measure to quantify model prediction performance (22, 23).

## Results

### Descriptive Analysis and Identification of Emerging Carcass Condemnation Reasons in California and the Rest of the US

**Figure 2:**
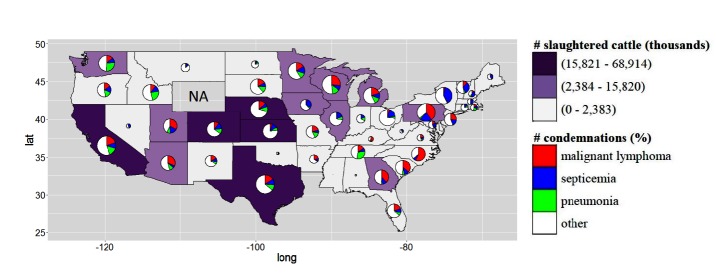
Number of the slaughtered cattle per state from Jan-2005 to Dec-2014 based on the 70, 90 and 100% percentile. The pie chart represents the percentage of the condemnation due to malignant lymphoma, septicaemia and pneumonia and other reasons of condemnation (red, blue, green and white, respectively). The size of the pie chart is computed as: log(number of condemnation carcasses)/10. NA = data not available. The accessible and dynamic version of this map is available in Disease BioPortal (http://bioportal.ucdavis.edu/).

**Figure 3:**
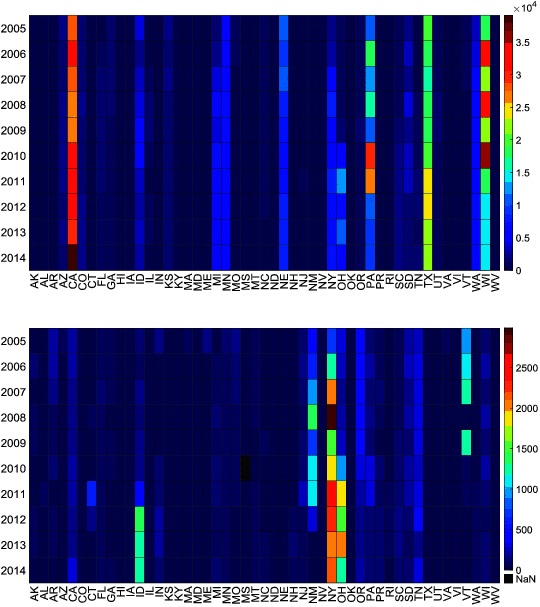
Number **(top)** and rate **(bottom)** of cattle carcasses condemned per state and per year in the US. The rate is in 10,000 cattle heads.

In the US a total of 1,442,745 carcasses were condemned at slaughterhouses across the US over the 10 year study period (Jan-2005 to Dec-2014). The most common condemnation reasons were malignant lymphoma followed by septicemia and pneumonia, all of them representing 39.6% of the US and 37.6% of the CA condemned carcasses, respectively (Figures 1, 2). Among all condemned carcasses in the US during that time period, California contributed approximately 50% of the cases of mastitis and 40% metritis cases and accounted for more than 20% of the condemned carcasses due to pericarditis, toxemia, pneumonia, peritonitis, abscess/pyemia, emaciation, nephritis/pyelitis, eosinophilic myositis, malignant lymphoma and miscellaneous inflammatory diseases. Although during this period, California was the main contributing state with 21% of total number of carcass condemnations in the US followed by Wisconsin with 16%, the highest rates of carcass condemnations were observed in New York and Ohio (Figure 3).

**Figure 4:**
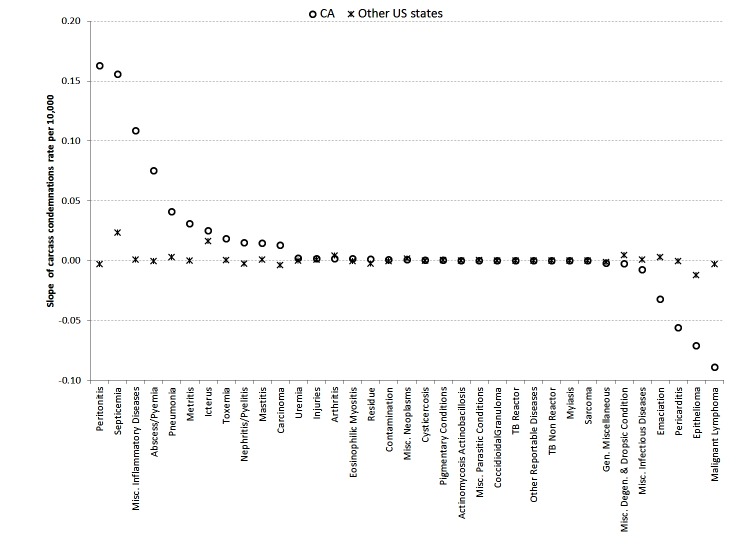
Calculated slope (based on integrated random walk model; Equation 5) for the rate of carcass condemnations (number of the condemned carcasses per reason/number of the slaughtered cattle heads) associated with different reasons from Jan-2005 to Dec-2014 in slaughterhouses in California (circle) and other US states (star). A positive calculated slope indicates an increase in the reported rate of carcass condemnations whereas negative slope indicates a decrease in the rate of carcass condemnations in slaughterhouses.

Results from the Random walk model with NVR = 0 are shown in Figure 4. The rate of cattle carcass condemnations per month associated with most of the condemnation reasons did not change (slope ~0) over the 10 year study period in both California and the rest of the US. The rate of cattle carcass condemnations associated with peritonitis, miscellaneous inflammatory diseases, abscess/pyemia, pneumonia, metritis, toxemia, nephritis/pyelitis, mastitis and carcinoma increased in California (slope >0) but remained stable (slope ~0) in the rest of US during the study period. Cattle condemnation rates associated with septicemia and icterus increased in both California and in the other US states. Cattle condemnation rates associated with epithelioma decreased in both California and other US states (slope <0); although the decrease in California was steeper than in the other US states. Miscellaneous infectious diseases, emaciation, pericarditis, malignant lymphoma decreased in California (slope <0) but remained stable in the other US states (slope ~0). Detail time series of all the cattle carcass condemnations are provided in the supplementary Figure S1 and Figure S2.

There were 35 different reasons for carcass condemnations recorded in slaughterhouses in California from Jan-2005 to Dec-2014 but only one slaughterhouse reported all 35 reasons. The main condemnation reasons in California were malignant lymphoma (20%), pneumonia (14%), septicemia (10%) and peritonitis (10%; Figure 1).

**Figure 5:**
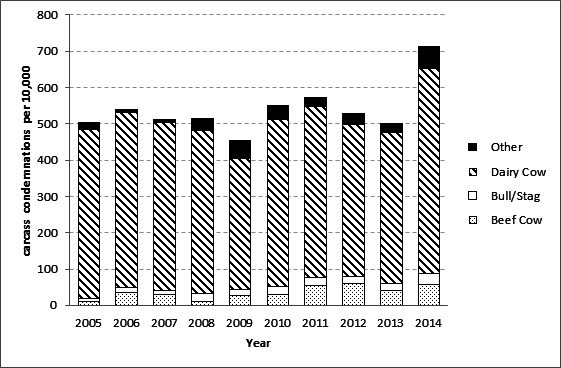
The rate of carcass condemnations per 10,000 animals per year (number of condemned carcasses per type of cattle i.e., dairy cow, bull/stag, beef cow and other)/number of cattle slaughtered per type of cattle) in California. The category of “other” type of cattle includes bob veal, formula-fed veal, heavy calf, heifer, non formula-fed veal and steer cattle.

There was an increase in the number of condemned carcasses in California from 2005 (27,556) to 2014 (39,101). Similarly, the number of slaughtered cattle in California increased from 2005 (1,356,302) to 2013 (1,734,792), but it decreased in 2014 (1,361,047), which lead to an increase in the rate of condemnations from 175 carcass per 10,000 in 2013 to 287 carcasses per 10,000 in 2014 (Figure 3). Interestingly, Tennessee was another state experiencing similar changes in the rate of carcass condemnations, with an increase from 174 carcass per 10,000 in 2013 to 285 carcass per 10,000 in 2014 (Figure 3). The main condemnation reasons contributing to the increased rate of carcass condemnations in California during 2014 were Pneumonia, Septicemia, Peritonitis, Abscess/Pyemia and Toxemia. Slaughtered dairy cows were more likely to be condemned due to malignant lymphoma, pneumonia, septicemia and peritonitis (99, 97, 70 and, 95% of the condemned carcasses, respectively) than beef cows in California (Table 1). Dairy cows and steers constitute 40 and 41%, respectively, of the total number of cattle sent to the slaughterhouses in California from Jan-2005 to Dec-2014, however, most (90%) of all cattle condemned in California from Jan-2005 to Dec-2014 were dairy cows (Figure 5, Table 1).

**Table 1:** Number of malignant lymphoma, abscess/pyemia, emaciation and, epithelioma carcasses condemned in California from Jan-2005 to Dec-2014 by type of cattle. The percentage is presented in parentheses.

**Type of cattle**	**Malignant Lymphoma**	**Pneumonia**	**Septicemia**	**Peritonitis**
Beef cow	316 (0.51)	262 (0.61)	481 (1.57)	97 (0.32)
Bob veal	18 (0.03)	146 (0.34)	7,982 (26.09)	914 (3.06)
Bull/Stag	72 (0.12)	64 (0.15)	104 (0.34)	58 (0.19)
Dairy cow	60,474 (98.71)	41,698 (96.68)	21,415 (70.00)	28,478 (95.38)
Formula-fed veal	0 (0)	4 (0)	0 (0)	1 (0)
Heavy calf	29 (0.05)	433 (1.00)	83 (0.27)	90 (0.30)
Heifer	88 (0.14)	181 (0.42)	129 (0.42)	83 (0.28)
Non formula fed veal	1 (0)	47 (0.10)	28 (0.09)	13 (0.04)
Steer	267 (0.44)	296 (0.69)	367 (1.20)	123 (0.41)
**Total**	**61,265**	**43,131**	**30,589**	**29,857**

### Modeling and Prediction of Temporal Dynamics of Cattle Carcass Condemnations in California From Jan 2005 to Dec 2014

**Figure 6:**
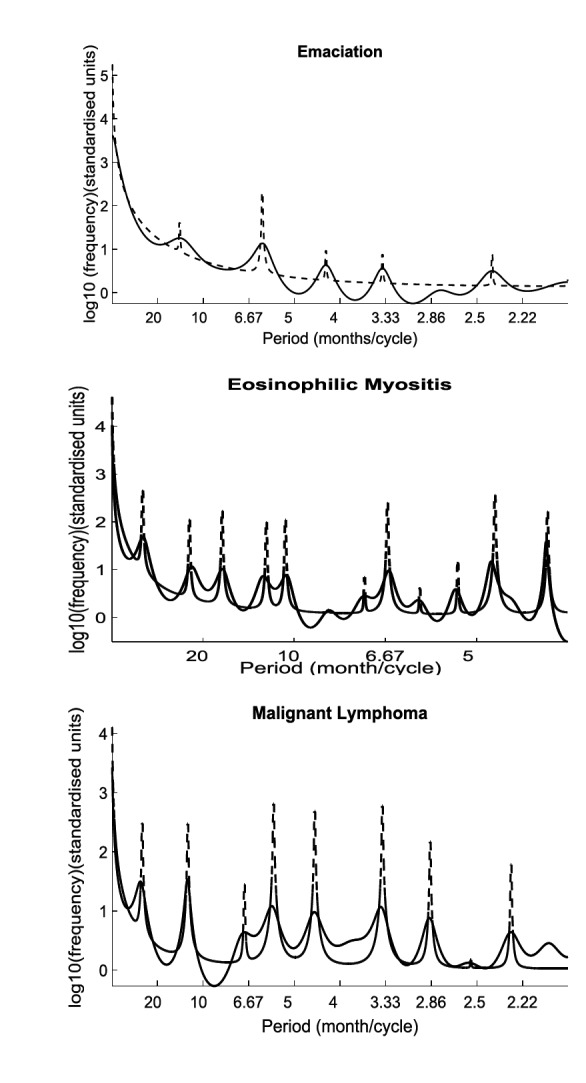
Autoregressive spectrum (23th order) of the reported emaciation (top), eosinophilic myositis (middle) and malignant lymphoma (bottom) condemnation cases in slaughterhouses in California. Solid line indicates the autoregressive spectrum of the carcass condemnation rate. The dashed line indicates the AR spectrum of the DHR model of the carcass condemnation rate using frequency domain NVR optimization.

**Figure 7:**
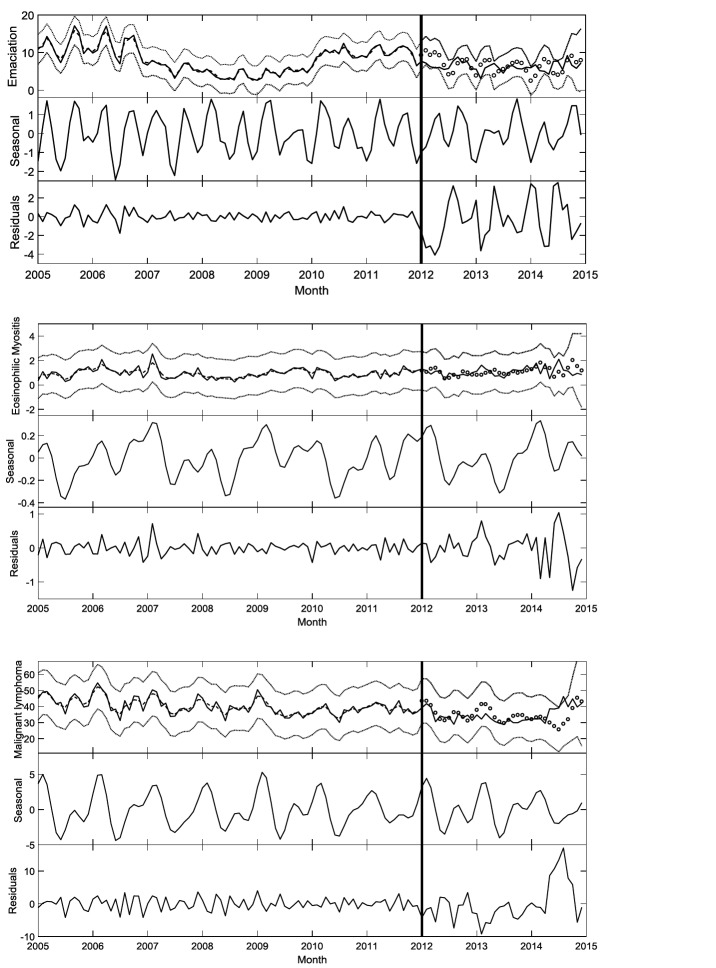
Results of the three Dynamic Harmonic Regression (DHR) models for the carcass condemnation rate [i.e., emaciation (top), eosinophilic myositis (middle) and malignant lymphoma (bottom)] in California from Jan-2005 to Dec-2014. The subplot at the top presents the model simulation results (in dashed line) from Jan-2005 to Dec-2011, the data (solid line) and the two times SE bounds (dotted lines) from Jan-2005 to Dec-2014 and, the 3 month ahead DHR model predictions from Jan-2012 to Dec-2014. The other two subplots present the seasonal component from Jan-2005 to Dec-2014 and the residuals extracted using the discrete-time white noise (Equation 1) from 2005 to Dec-2011 and the prediction error from Jan-2012 to Dec-2014

Based on the autoregressive spectrum results in California, only the rate of cattle carcass condemnations associated with emaciation, eosinophilic myositis, and malignant lymphoma showed seasonal components (Figure 6). Therefore, three predictive DHR models were built to detect the annual frequencies of emaciation, eosinophilic myositis, and malignant lymphoma. The trend estimate, forecasts of the series (with ± two times SE), seasonal component and the remaining residual components for the three condemnation reasons, are all illustrated in Figure 7.

The rate of condemnations associated with emaciation showed pronounced peaks at periods of 13.3, 6.1, 4.3, 2.5 and 2.1 (month/cycle), with clear increase in spring and fall and decrease in summer Figures 6 top and 7). The rates associated with eosinophilic myositis showed both multiannual and seasonal periodic component by the main pronounced peaks at 29.5, 11.8, 8.3, 6.8, 5.9, 2.6 and 2.4 (month/cycle) that declined in spring and peaked in summer and winter (Figures 6 middle and 7). The malignant lymphoma time series contained a 12 month periodicity and multiannual fluctuations with pronounced peaks at periods of 29.9, 12, 6.9, 5.6, 4.5, 3.4, 2.5 and 2.0 (month/cycle) with an increase observed during the winter (Figures 6 bottom and 7).

**Figure 8:**
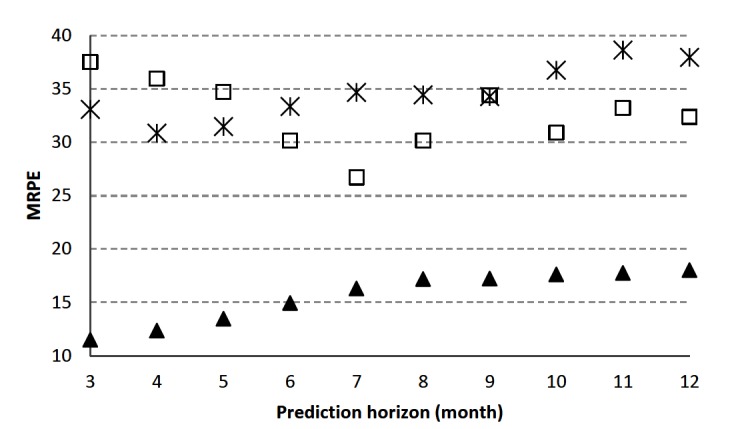
Mean relative prediction error (MRPE) vs prediction horizon for the rate of carcass condemnations of emaciation (star), eosinophilic myositis (square) and malignant lymphoma (triangle) in California. The smaller MRPE indicates a closer fit of the model to the data.

The DHR model simulated the dynamics of the reported rate of emaciation, malignant lymphoma and eosinophilic myositis from Jan-2005 to Dec-2011 with an $R_{T}^{2}$ of 0.98, 0.87, and 0.78, respectively, and could predict the rate of condemned carcasses three months ahead from Jan-2012 to Dec-2014 with MRPE of 33, 11, and 38% for the three carcass condemnation reasons, respectively (Figure 8). The MRPE was used to compare the model performance for a range of prediction horizons (from 3–12 month, Figure 8). The rate of the carcass condemnation can be predicted 12 month ahead with the MRPE of 38, 18 and 32% for emaciation, malignant lymphoma and eosinophilic myositis, respectively (Figure 8).

## Discussion

In this study we analyzed slaughterhouse data from the US to show the value of the systematic analysis of slaughter data and to describe the most important condemnation reasons in the US and California, identify emerging condemnation reasons, characterize seasonality and temporal patterns and predict the rate of carcass condemnations in different time horizons (i.e., from 3 to 12 months ahead). We believe that the continuous and systematic analysis of slaughter data as shown here can lead to establishment of a low-cost monitoring system for early detection of emerging problems and zoonotic diseases in food animals.

The rate of carcass condemnations associated with septicemia and icterus showed clear evidence of increase both in California and in the rest of the US. Peritonitis, misc. inflammatory diseases, abscess/pyemia, pneumonia and metritis were increasing only in California (Figure 4). The emergence of these condemnations could be the result of a number of impacts in all parts of the food chain, such as environmental and management conditions, stress, pathogens, etc. (24). Because the state of origin of the cattle may be different from the location of the slaughterhouse, a better understanding of the patterns of carcass condemnations together with the successful trace back of any diagnostic cases found in the slaughterhouse could help identify where an animal may have acquired infection, the location of any other animals that may have been exposed to the same pathogens, or find locations that share risk factors or management practices that may lead to carcass condemnation.

The USDA FSIS data from 2005 to 2015 showed that the cull dairy cows in CA were more likely to be condemned for malignant lymphoma, pneumonia, septicemia and peritonitis than the beef cows (Table 1). This disproportion of condemned carcasses among the type of cattle is due to the fact that the priorities in the dairy industry are milk production, herd reproduction, and prevention of health conditions such as mastitis (25) and dairy producers also have a number of other issues that might compete with their need to change culling policies (2). In general the production system in dairy cattle is intense and their lifespan is long (around 3–4 years) therefore there is more chance for dairy cattle to develop age related problems or chronic diseases such as Johne’s disease or cancer that can cause emaciation and other deteriorating symptoms in the animals.

Seasonality was found to be significant only in three condemnation reasons in California: emaciation, eosinophilic myositis and malignant lymphoma (Figures 6 and 7), which are representing 3.9, 0.5 and 19.9% of total condemned carcasses in California respectively. The seasonal variation in the number of condemned carcasses could be linked to management and environmental factors that vary seasonally (e.g., market costs, land constraints, or production goals), or to the seasonal occurrence of diseases or syndromes that lead to carcass condemnation. A study in Ontario from 2001 to 2007 (5) found seasonal effects for the number of condemned carcasses showing fewer condemned carcasses in the summer and fall compared to winter. Vial and Reist et al. (3) found seasonal patterns in the number of carcass condemnations in cattle in Switzerland between 2007 and 2012 with a peak in March. A peak in the number of condemned cattle can also occur when more animals are sent to slaughter or when lower quality animals are sent to slaughter in specific seasons. Seasonality could also be associated with forage availability and feed prices among other seasonal changes in management practices and in the environment that have a direct impact animal health (3, 5).

Eosinophilic myositis exact causes are unknown although it has been associated with Sarcocystis cruzi infection (26). A more refined spatial analysis and trace back of where those carcasses are coming from may help to identify high risk areas where particular attention and biosecurity measures should be taken to avoid *S. cruzi* infection in cattle.

Emaciation is associated with wasting conditions caused by chronic diseases. Known causes of emaciation are parasitism, chronic abscess, chronic musculoskeletal pain, advance neoplasias, Johne’s disease, and malnutrition (27). Dairy cows contribute most to the number of condemned carcasses in California for these conditions (85%). Our study shows seasonality in the number of condemned carcasses due to emaciation, with a peak in spring and fall and a drop in summer (Figure 7). The observed seasonality most likely has to do with the number and type of cows culled in the fall that put adult cows that were not pregnant during the calving season in spring are more likely to be culled in fall after four or five unsuccessful breeding attempts (28).

Malignant lymphoma is the clinical manifestation of an infection caused by the retrovirus bovine leukemia virus (BLV), which infects cattle’s lymphoid tissue. Once in the herd, the virus can be transmitted by blood containing infected lymphocytes in shared needles and during dehorning, tattooing or rectal examination (29). When tumors are found at slaughter, carcasses are condemned following federal regulation (30). Malignant lymphoma is the main cause of carcass condemnation in California and other US states (Figure 1) and it affects mainly dairy cattle, which contributed to 99% of the all malignant lymphoma condemned carcasses in California from Jan-2005 to Dec-2014 (Table 1). In our current study, we found a declining trend in the rate of condemned carcasses due to malignant lymphoma in California. Two previous studies in 1996 and 2007 USDA dairy studies showed a slight decrease in prevalence from 89.0 to 83.9% seropositive dairy herds due to BLV (31). However, the 2007 dairy study also showed higher prevalence in the East (84.4%) than West (78.4%), which could explain the different trends observed between California and the rest of US. Condemned carcasses due to malignant lymphoma peak in winter. Because BLV is a chronic disease, the peak is most likely associated with management decisions. In addition, BLV affects the immune system (making cattle more susceptible to other diseases), and when this is combined with winter environmental factors, cows can be clinically poorer in winter and have decreased milk production, resulting in the cattle being sent to slaughter. Also, since biting insects can transmit BLV (32), changing environmental conditions during the study period can have an impact in insect ecology and disease dynamics. Since distribution of infection is uneven across farms within a region(29), detailed slaughterhouse data with possibility to trace back those cases to the farm of origin could help approximate BLV prevalence in different areas in California where BLV has a big impact on the dairy industry.

In this study we also aimed to evaluate the predictive ability of our models for different time horizons (3–12 month). Therefore, we quantified the model performance predicting the rate of carcass condemnations due to emaciation, malignant lymphoma and eosinophilic myositis from Jan-2012 to Dec-2014. A better understanding of the expected rate of condemnation cases by state, slaughterhouse and farm can definitely help producers and veterinary practitioners better plan and optimize management and disease control strategies to mitigate causes leading to those condemnations. Our results illustrate that the model predicts the rate of carcass condemnations due to emaciation, eosinophilic myositis, and malignant lymphoma quite well for one season (3 months) and up to 12 months ahead with a MRPE of 33, 38 and 11%, respectively (Figure 8). Although the MRPE values in our study were not very low, they were around 10% lower than values found by other researchers for similar modeling approaches in similar applications (18). Similarly, the $R_{T}^{2}$ values in our study were considerably high ($R_{T}^{2}$ of 0.98, 0.78 and 0.87 for emaciation, eosinophilic myositis, and malignant lymphoma, respectively) showing a good model fit.

It should be noted that our models did not use any variables as inputs in the data-based model. Taking into account management and climatic factors are expected to further improve the modeling results, although the dynamics of the emaciation, malignant lymphoma and eosinophilic myositis condemned carcasses in slaughterhouses in California were already predicted satisfactorily just based on the cyclic components of the time series. The specific role of environmental and climatic factors in the dynamics of the reasons for carcass condemnation is an ongoing area of research. A dataset covering a longer period might also improve the modeling results. Having access to information on the total animals slaughtered in each slaughterhouse per day, instead of just aggregated by month and by state, will allow us to conduct more detailed spatio-temporal analyses and generating semi-automatic notifications/alerts when the observed number of condemned carcasses exceeds the expected condemned carcasses in a particular slaughterhouse/area. Future studies may also be conducted to understand and predict how long-term environmental and climatic trends (e.g., factors associated with climatic change) and their variability may impact cattle health, animal welfare and livestock production sustainability.

## Conclusions

Slaughter data is a valuable source of information for animal and public health. Our DHR modeling approach provided valuable insights to identify emerging syndromes in the US and quantify the inter-annual changes in the trend and seasonality of the rate of carcass condemnations associated with emaciation, malignant lymphoma and eosinophilic myositis in cattle slaughterhouses in California. Such a data-based modeling approach could be easily extended to other species and can provide the foundations to develop a low-cost syndromic surveillance system using real-time slaughterhouse information to early-detect outbreaks or changes in the number of condemned carcasses in specific locations and time periods. This could be very useful to inform producers and veterinary practitioners to improve farm level management practices and interventions to minimize and prevent the drivers contributing to those condemnations, saving producers and livestock industry millions of US dollars annually.

## Author Contributions

BM-L and SAH conceived and designed the study. SAH processed the data and conducted the analyses under the supervision of BM-L. BM-L, GV, AE, DM and NSR contributed to the critical interpretation and discussion of the results. All authors read, reviewed, and approved the final manuscript.

## Conflict of Interest Statement

The authors declare that the research was conducted in the absence of any commercial or financial relationships that could be construed as a potential conflict of interest.

## Supplementary material

Figure S1 Time series of the carcass condemnation rates (number of condemned carcasses/ number of cattle slaughtered; solid line) observed in California from 2005-2014 with the trend line (bold line) and two times standard error bounds (dotted lines) for each of the 35 condemnation reasons.

Figure S2 Time series of the carcass condemnation rates (number of condemned carcasses/ number of cattle slaughtered; solid line) observed in the other US states (without California) from 2005-2014 with the trend line (bold line) and two times standard error bounds (dotted lines) for each of the 35 condemnation reasons.
