# Supplementary figures and images for: Characterization of the Temporal Trends in the Rate of Cattle Carcass Condemnations in the US and Dynamic Modeling of the Condemnation Reasons in California With a Seasonal Component
Source: Front Vet Sci. 2018 Jun 19;5:87. doi: 10.3389/fvets.2018.00087 (PMC6018506; doi:10.3389/fvets.2018.00087)

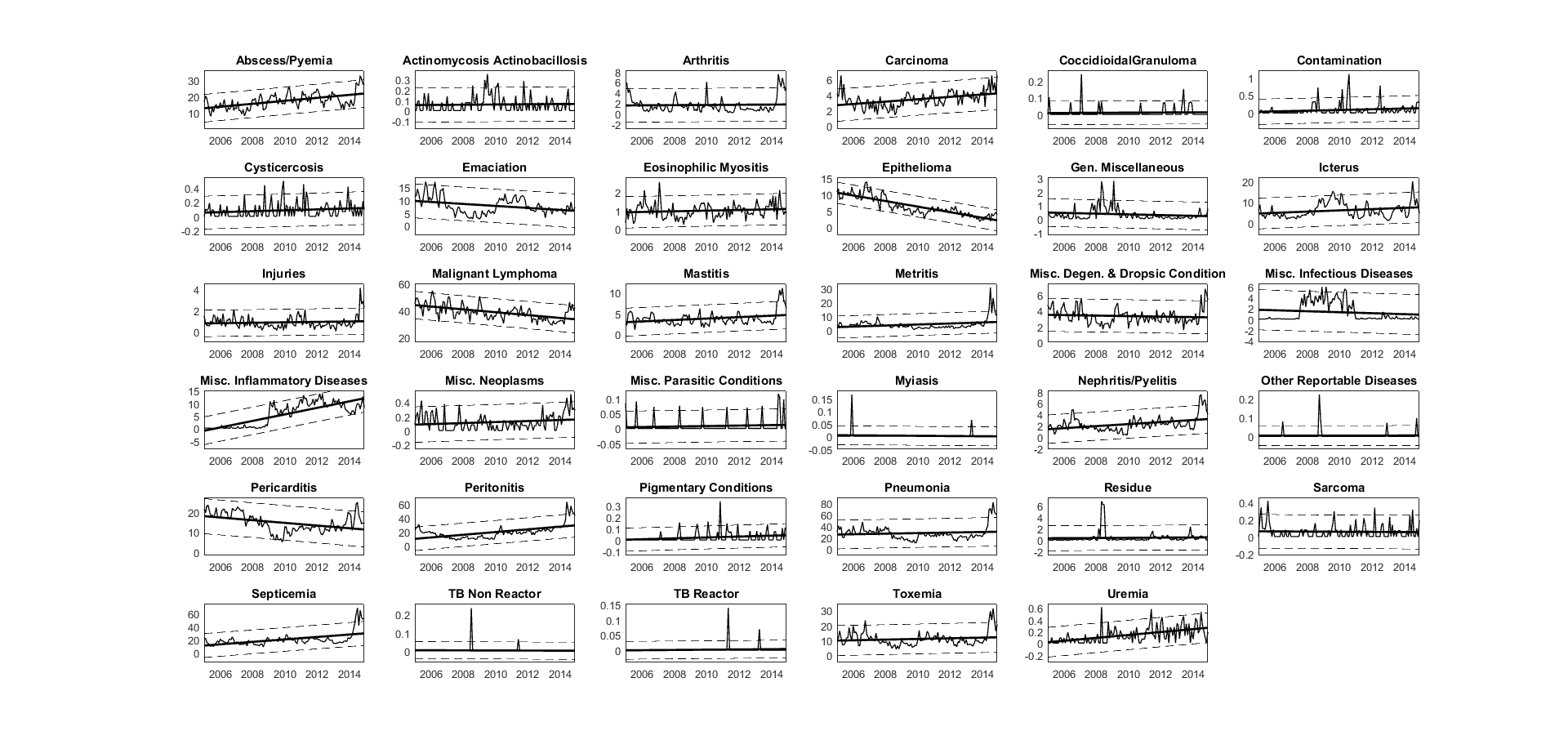

Supplement: Figure S1 — Time series of the carcass condemnation rates (number of condemned carcasses/ number of cattle slaughtered; solid line) observed in California from 2005-2014 with the trend line (bold line) and two times standard error bounds (dotted lines) for each of the 35 condemnation reasons. [file Image1.TIF]

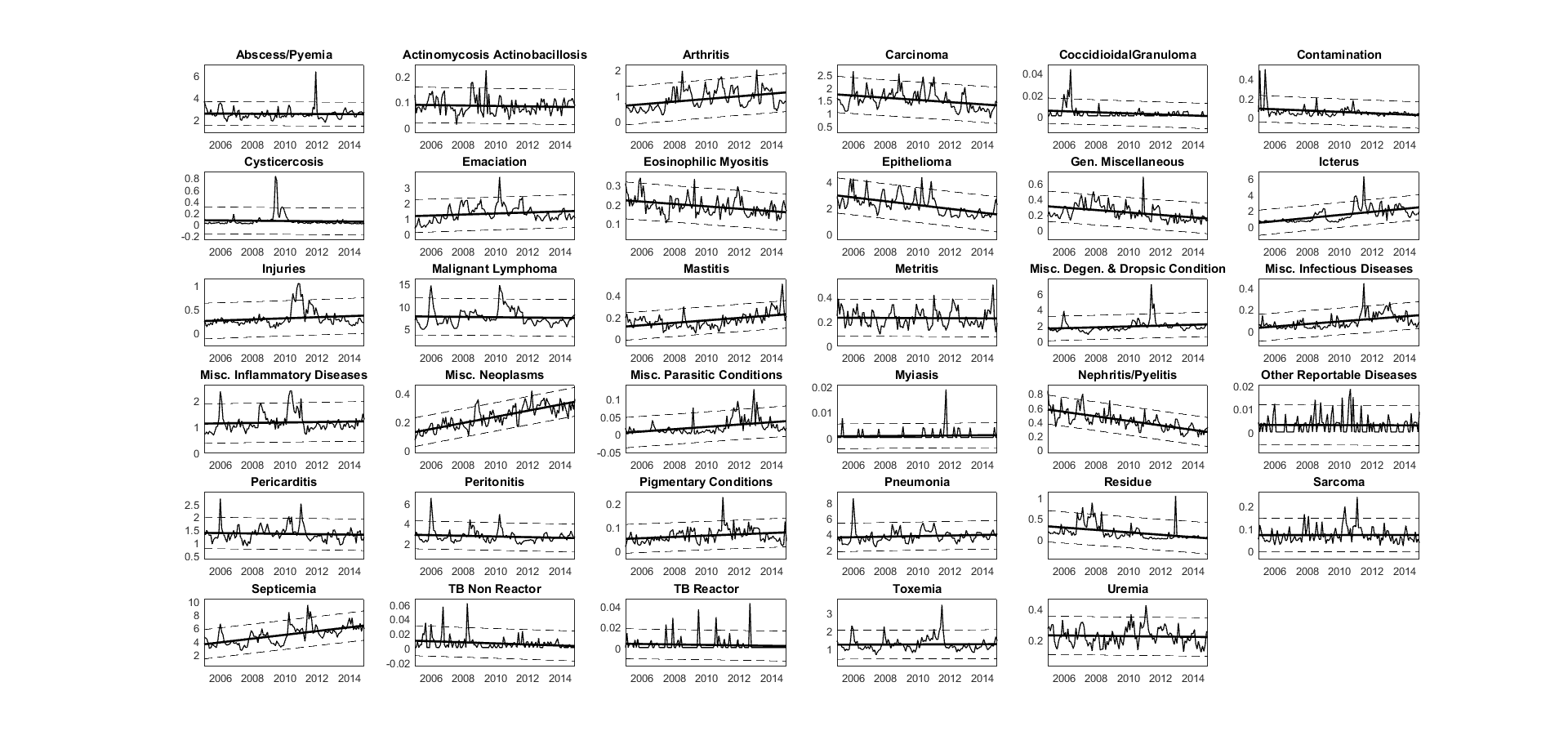

Supplement: Figure S2 — Time series of the carcass condemnation rates (number of condemned carcasses/ number of cattle slaughtered; solid line) observed in the other US states (without California) from 2005-2014 with the trend line (bold line) and two times standard error bounds (dotted lines) for each of the 35 condemnation reasons. [file Image2.TIF]
